# Clinical Outcomes of Permanent Left Bundle Branch Area Pacing in Patients With Left Bundle Branch Block and Left Ventricular Ejection Fraction >35 vs. ≤35%

**DOI:** 10.3389/fcvm.2022.838708

**Published:** 2022-03-17

**Authors:** Zhixin Jiang, Tian Wu, Yixian Wu, Zenghong Chen, Wen Yang, Chongchong Chen, Xiujuan Zhou, Qijun Shan

**Affiliations:** Department of Cardiology, Jiangsu Province Hospital, The First Affiliated Hospital of Nanjing Medical University, Nanjing, China

**Keywords:** left bundle branch block, left bundle branch area pacing, cardiac resynchronization therapy, QRS duration, heart failure

## Abstract

**Aims:**

The present study aimed to compare the effects of left bundle branch area pacing (LBBAP) on cardiac function and clinical outcomes in patients with left bundle branch block (LBBB) and left ventricular ejection fraction (LVEF) >35 vs. ≤35%.

**Methods and Results:**

Thirty-six consecutive patients with LBBB and LVEF <50% were enrolled. All patients were followed up for a mean of 6 months. The successful LBBAP was defined as a paced QRS complex presented as right bundle branch block (RBBB) morphology and QRSd < 130 ms. Echocardiography parameters, pacing parameters and clinical outcomes were collected. The successful LBBAP was achieved in 77.8% of all cases (28/36). In LVEF > 35% group (70 ± 8 years, 9 male), the success rate was 81.0% (17/21). QRSd significantly decreased from 174 ± 23 ms to 108 ± 13 ms (*P* < 0.001). The pacing threshold and R-wave amplitude were 0.6 ± 0.2 V @ 0.5 ms and 12 ± 7 mV, respectively. In LVEF ≤ 35% group (69 ± 5 years, 9 male), the success rate was 73.3% (11/15) with QRSd decreasing from 188 ± 25 ms to 107 ± 11 ms (*P* < 0.001). The hyperresponders to LBBAP (functional recovery and LVEF ≥ 50%) in LVEF > 35% group was 52.9%, which were almost twice of that in LVEF ≤ 35% group (33.3%). Whether patients had LBBAP or left ventricular septal pacing (LVSP), patients in the LVEF > 35% group showed significantly lower incidence of heart failure hospitalizations or death from any cause (hazard ratio in LVEF > 35% group, 0.22; 95%CI, 0.06 to 0.75, *P* = 0.011).

**Conclusions:**

LBBAP can significantly shorten the QRSd and improve cardiac function in LBBB patients with either LVEF > 35 or ≤ 35%. LBBAP should be considered as an effective therapy for preventing the deterioration of cardiac function in early-stage heart failure patients with LBBB and LVEF > 35%.

## Introduction

It is well established that left bundle branch block (LBBB) has bad effect on left ventricular (LV) function independent of coexisting heart disease. The electromechanical dyssynchrony of the ventricular contractions can contribute to adverse remodeling, reduction of left ventricular ejection fraction (LVEF), and mitral regurgitation in the long term. Cardiac resynchronization therapy (CRT), which involves simultaneous pacing of both right and left ventricles is beneficial and widely used around the world. Major US (ACC/AHA/HRS) (1) and European Society of Cardiology (ESC) guidelines (2, 3) were consistent in issuing Class I and IIA recommendations for CRT in patients who have LVEF ≤ 35% and LBBB with a QRS duration (QRSd) ≥ 150 ms, and New York Heart Association (NYHA) class II, III, or ambulatory IV symptoms. However, when LVEF is more than 35%, the recommendation level degrades, which seems to be arbitrary. The LVEF cut-off of ≤ 35% is adopted by heart failure (HF) major clinical trials of CRT, such as COMPANION (4), because people with LVEF ≤ 35% have higher incidence of adverse events, both in terms of sensitivity and specificity of incidence. However, LVEF or LV systolic dysfunction are continuous variables. And LVEF measured by echocardiography is not highly precise compared to magnetic resonance imaging (MRI). In addition, patients with LVEF > 35% are being neglected and the proportion of them is increasing. And they have similar characteristics and treatment patterns to those with LVEF <35%. In eraly-stage HF patients, those with LBBB have significantly worse clinical outcomes than patients without conduction system disease. Although common practice indicates that we implant CRT outside of guideline recommendations, randomized, multicenter studies in this population have not been conducted yet.

What's more, up to 30% of patients do not respond to CRT and the published data may be underestimated (5). Significant scar burden related to lead position (6), QRSd <150 ms (7), right ventricular failure (8), right bundle branch block (RBBB) morphology (9) have been demonstrated to be associated with lack of response. And in combination with national conditions of China, the price of CRT may be too high to be accepted in many patients. In 2017, Huang et al. (10) first reported a novel pacing method to correct the LBBB in the site of the left bundle branch (LBB) area with low and stable output; clinical outcome significantly improved over one year of follow-up. A large single center study (11), which included 632 patients who underwent left bundle branch area pacing (LBBAP), demonstrated that LBBAP was feasible and safe with high success rate in bradycardia or HF patients during long-term follow-up. And several studies (12, 13) have proved that LBBAP could achieve narrowing of QRS duration and improvement of clinical and echocardiographic outcomes in HF patients with LBBB, which means that LBBAP could be a promising resynchronization therapy alternative to biventricular pacing (BVP) for patients with CRT-indications. Since LBBAP is more convenient and cheaper compared to CRT, it would be of clinical interest whether LBBAP could benefit for the HF patients with LBBB and LVEF > 35%. Consequently, this study was undertaken to compare the clinical outcomes of LBBAP in patients with LBBB and LVEF > 35 vs. ≤ 35%.

## Methods

### Study Population

This was a single-center retrospective study. Consecutive patients underwent LBBAP were enrolled from the First Affiliated Hospital of Nanjing Medical University between May 2017 and December 2020. Patients who met the following criteria were included: (1) complete LBBB morphology that met Strauss criteria (14); (2)echocardiographic evidence of LVEF < 50%; (3) follow-up period over 6 months. All the patients included were provided written informed consent to the study protocol, and were approved by the Institutional Review Board.

### Implantation Procedure

The technique of LBBAP procedure has been described in previous reports (15–19). Briefly, a ventricular pacing electrode (Medtronic 3830 electrode) with a 7-Fr guiding catheter (Model C315-S10; Medtronic Inc) was introduced into right ventricle via left subclavian or axillary vein, from His bundle area advanced 1–2 cm toward the right ventricle apex against the ventricular septum, then screwed through the interventricular septum (IVS) to the LBB area. When unipolar paced QRS complex presented as right bundle branch block morphology (qR or rSR' morphology in V1), and pacing parameters were satisfied, lead position and no perforation were assessed by angiogram through C315 sheath under left anterior oblique (LAO) 40°, the sheath was removed and lead was fixed. Successful LBBAP was defined as unipolar paced QRS morphology present as RBBB pattern and QRSd < 130 ms (15). If successful LBBAP could not be achieved after 5 attempts of lead positioning or fluoroscopy duration exceeded 30 min, the left ventricular septum pacing (LVSP) was also accepted, placing the 3,830 lead in the LV mid-septum to achieve a relatively narrow QRSd (17).

### Data Collection

The baseline characteristics and medical history of participants were collected at enrollment. The LBBAP paced electrocardiogram (ECG) were interpreted by two cardiologists. The stimulus to peak LV activation time (SPLVAT), defined as the duration between the ventricular stimulation signal and R peak in lead V5, was measured, which meant LBBAP indirectly captured either the main LBB or its branches as previously described (15, 17, 18). Other electrocardiographic parameters such as the intrinsic QRSd, paced QRSd (pQRSd) were also measured. Pacing parameters like pacing thresholds, pacing impedance, R-wave amplitude were recorded. Echocardiographic parameters including left atrial dimension (LAD), left ventricular end diastolic diameter (LVEDD), left ventricular end systolic diameter (LVESD) and LVEF were also recorded.

### Follow Up

Patients were followed up in the clinic or in hospital at baseline, 3, 6 and 12 months. Clinical characteristics, echocardiographic parameters and lead-related complications were recorded. LBBAP responder was defined as a patient who had an LVEF improvement of at least 5% at the 6-month follow-up. Patients were considered to be “hyperresponders” (20), if they met two following criteria: functional recovery and LVEF ≥ 50%. The primary composite endpoint included death from any cause or hospitalizations for HF. The diagnosis of HF hospitalization was made by professional physicians, if patients were developing symptoms that current treatments could not control and have to be hospitalized again due to the congestive HF.

### Statistical Analysis

Continuous variables were expressed as mean ± standard deviation (SD). Categorical variables were expressed as numbers and percentage values and compared using chi-square or Fisher's exact test. Comparisons between continuous variables were tested using Student's *t*-test. Kaplan–Meier survival curves were used to estimate for the combined endpoint of time to death or first HF hospitalization. The log rank test compared survival curves between two groups. Statistical analysis was performed using SPSS version 20.0 software. All *P*-values were two-tailed and *P*-values < 0.05 were considered significant.

## Results

### Baseline Characteristics

From May 2017 to December 2020, 496 patients underwent LBBAP. Of the 69 patients who had complete LBBB morphology, 33 patients with normal LVEF were excluded. Finally, 36 LBBB patients (age: 70 ± 7 years, male = 18) underwent an attempted LBBAP. There were 15 LBBB patients with LVEF ≤ 35% (27.9 ± 4.7), 21 LBBB patients with LVEF > 35% (40.2 ± 4.5). Successful LBBAP was achieved in 77.8% (28/36) of cases. The baseline characteristics were summarized in Table 1. There was no difference in age, sex, QRSd, drug utilization and complications including hypertension, diabetes mellitus, renal insufficiency, syncope, coronary artery disease and atrial fibrillation (AF) between the two groups. The LAD, LVEDD and LVESD were significantly higher in LVEF ≤ 35% group (46 ± 8 vs. 41 ± 7, *P* < 0.05; 72 ± 9 vs. 58 ± 6, *P* < 0.001; 63 ± 8 vs. 46 ± 5, *P* < 0.001).

**Table 1:** Baseline characteristics.

	**All patients**	**LVEF ≤35%**	**LVEF >35%**	***P*–value**
	**(*n =* 36)**	**(*n =* 15)**	**(*n =* 21)**	
LBBAP success rate (%)	28 (77.8)	11 (73.3)	17 (81.0)	0.69
Age (years)	70 ± 7	69 ± 5	70 ± 8	0.55
Male (%)	18 (50)	9 (60.0)	9 (42.9)	0.50
QRS duration (ms)	180 ± 25	188 ± 25	174 ± 23	0.10
Hypertension (%)	23 (63.9)	7 (46.7)	16 (76.2)	0.09
Diabetes mellitus (%)	8 (22.2)	2 (13.3)	6 (28.6)	0.42
Renal insufficiency (%)	8 (22.2)	5 (33.3)	3 (14.3)	0.24
Syncope (%)	3 (8.3)	2 (13.3)	1 (4.8)	0.56
Coronary artery disease (%)	10 (27.8)	4 (26.7)	6 (28.6)	1.00
Paroxysmal AF (%)	4 (11.1)	2 (13.3)	2 (9.5)	1.00
Persistent AF (%)	6 (16.7)	2 (13.3)	4 (19.0)	1.00
Beta-blocker (%)	35 (97.2)	15 (100.0)	20 (95.2)	1.00
ACE inhibitor/ARB (%)	16 (44.4)	7 (46.7)	9 (42.9)	1.00
Diuretics (%)	29 (80.6)	13 (86.7)	16 (76.2)	0.67
Digitalis (%)	10 (27.8)	6 (40.0)	4 (19.0)	0.26
Sacubitril valsartan (%)	26 (72.2)	11 (73.3)	15 (71.4)	1.00
dapagliflozin (%)	6 (16.7)	2 (13.3)	4 (19.0)	1.00
LAD (mm)	43 ± 8	46 ± 8	41 ± 7	0.04
LVEDD (mm)	64 ± 10	72 ± 9	58 ± 6	<0.001
LVESD (mm)	53 ± 10	63 ± 8	46 ± 5	<0.001
LVEF (%)	35.1 ± 7.6	27.9 ± 4.7	40.2 ± 4.5	<0.001

*AF, atrial fibrillation; ACE, angiotensin converti enzyme; ARB, angiotensin receptor blocker; LAD, left atrial dimension; LVEDD, left ventricular end–diastolic dimension; LVESD, left ventricular end–systolic dimension; LVEF, left ventricular ejection fraction*.

### Pacing Parameters

In LVEF ≤ 35% group, the QRSd significantly decreased from 188 ± 25 ms to 107 ± 11 ms (*P* < 0.001) and the SPLVAT was 88 ± 13 ms. In LVEF > 35% group, the QRSd also decreased from 174 ± 23 ms to 108 ± 13 ms (*P* < 0.001), and the SPLVAT was 88 ± 15 ms. In Table 2, during the LBBAP procedure, R-wave amplitude of LVEF > 35% group was significantly higher than LVEF ≤ 35% group (12 ± 7 mV vs. 7 ± 3 mV, *P* < 0.01). The pacing threshold, pacing impedance, paced QRSd and SPLVAT between the two groups were of no significance.

**Table 2:** Pacing parameters in successful LBBAP patients.

	**LVEF ≤35%**	**LVEF >35%**	***P-*value**
	**(*n =* 11)**	**(*n =* 17)**	
Pacing threshold (V/0.5ms)	0.9 ± 0.4	0.6 ± 0.2	0.08
R–wave amplitude (mV)	7 ± 3	12 ± 7	0.008
Pacing impedance (Ω)	661 ± 112	709 ± 127	0.32
Paced QRS duration (ms)	107 ± 11	108 ± 13	0.73
SPLVAT (ms)	88 ± 13	88 ± 15	1.00

*SPLVAT, stimulus peak to left ventricular activation time*.

### Clinical Outcomes

**Figure 1:**
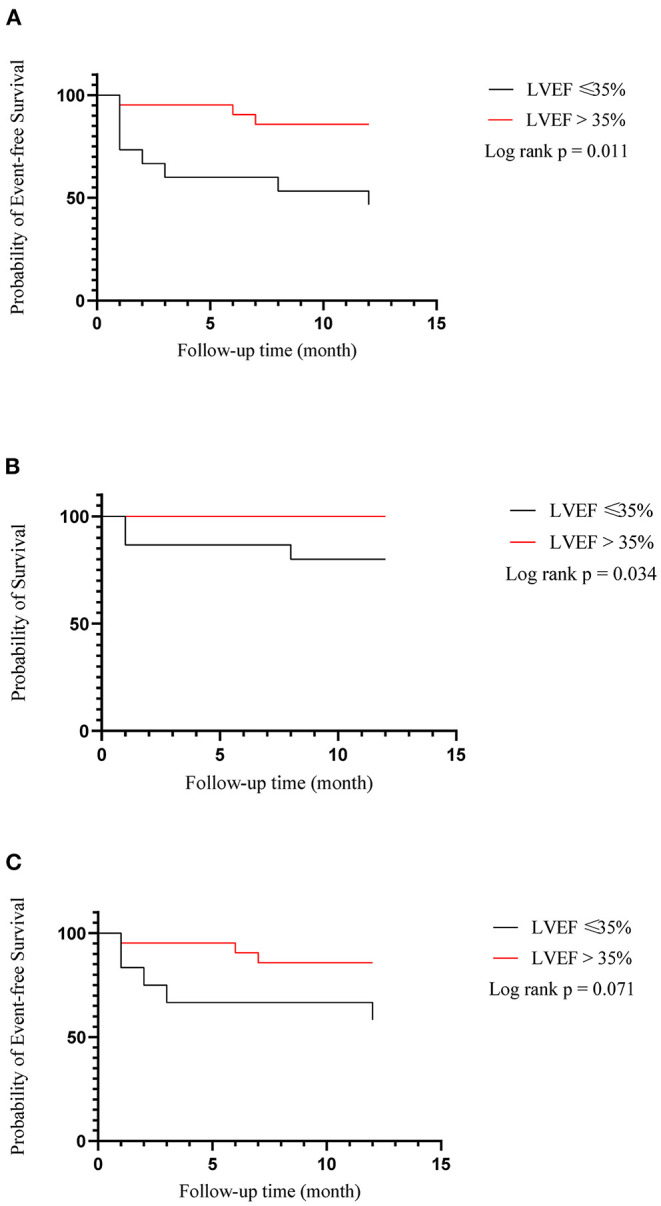
Kaplan–meier estimates of death or hospitalization for heart failure (composite primary outcome), death from any cause and hospitalization for heart failure among all of 36 patients recruited. **(A)** Heart failure hospitalization or death from any cause; **(B)** Death from any cause; **(C)** Heart failure hospitalization.

During the follow-up of a mean of 6 months, no complications associated with LBBAP such as lead perforation and dislodgement, pericardial effusion, pneumothorax, and thromboembolism were observed. There was one person in each group who had pocket infection and underwent incision and drainage of pocket. Clinical endpoint in successful LBBAP patients at the 12-month follow-up was shown in Table 3. The primary outcome occurred in 3 of 17 patients (17.6%) in LVEF > 35% group and 5 of 11 patients (45.5%) in LVEF ≤ 35% group. In addition, as shown in Figure 1, in all 36 patients recruited, the Kaplan-Meier survival curve of the primary endpoint of LVEF > 35% group including hospitalization for HF or death from any cause was significantly higher than of LVEF ≤ 35% group (hazard ratio in LVEF > 35% group, 0.22; 95%CI, 0.06 to 0.74, *P* = 0.011). And so did the Kaplan-Meier survival curve of death from any cause with *P*-value < 0.05.

**Table 3:** Clinical endpoints in successful LBBAP patients at the 12-month follow-up.

	**LVEF ≤35%**	**LVEF >35%**	***P-*value**
	**(*n =* 11)**	**(*n =* 17)**	
Death (%)	2 (18.2)	0 (0)	0.07
Heart failure hospitalization (%)	3 (27.3)	3 (17.6)	0.32
The primary composite endpoint (%)	5 (45.5)	3 (17.6)	0.10

**Figure 2:**
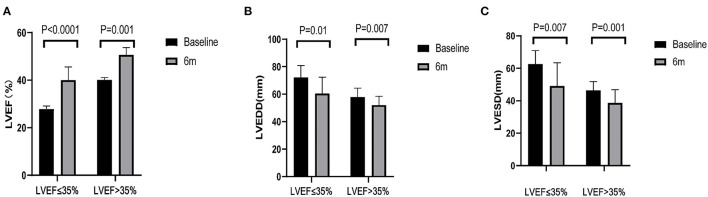
Echocardiographic parameters. **(A–C)** Left ventricular ejection fraction (LVEF), left ventricular end-diastolic dimension (LVEDD) and left ventricular end-systolic dimension (LVESD) of patients pre-left bundle branch area pacing (LBBAP) and 6 months after LBBAP.

As it was shown in Table 4 and Figure 2, LAD, LVEDD, LVESD had shortened and LVEF had improved in both groups, but there was no difference in ΔLAD, ΔLVEDD, ΔLVESD and ΔLVEF between the two groups. However, the number of LBBAP hyperresponders in LVEF > 35% group was 9 (52.9%), which was almost twice of that in LVEF ≤ 35% group (33.3%).

**Table 4:** LBBAP response and clinical outcomes at 6-month follow-up.

	**LVEF ≤35%**	**LVEF >35%**	***P-*value**
	**(*n =* 9)**	**(*n =* 17)**	
LVEF decrease	1 (11.1)	3 (17.6)	1.00
LVEF improve <5%	3 (33.3)	3 (17.6)	0.63
LVEF improve ≥5%	2 (22.2)	2 (11.8)	0.59
LVEF ≥50% (hyperresponders)	3 (33.3)	9 (52.9)	0.43
Change in LAD	−1.8 ± 5.6	−2.5 ± 3.8	0.75
Change in LVEDD	−9.3 ± 8.6	−5.8 ± 6.2	0.24
Change in LVESD	−11.7 ± 11.0	−7.6 ± 8.5	0.30
Change in LVEF	12.6 ± 14.9	10.2 ± 13.4	0.68

*LAD, left atrial dimension; LVEDD, left ventricular end-diastolic dimension; LVESD, left ventricular end-systolic dimension; LVEF, left ventricular ejection fraction. Two patients in LVEF ≤ 35% group died in the follow-up with no echocardiography recorded*.

In our study, a total of 3 patients died (one at 8 month, two at 1 month) after LBBAP due to progressive HF. The baseline LVEF of these patients were below 35%. Of them, 2 accepted LBBAP and 1 accepted LVSP. They were over 70 years old and had HF for many years with N-terminal pro-B-type natriuretic peptide (NT-proBNP) over 7000 pg/ml before LBBAP. Two of them had chronic kidney disease (21), which is a major contributor to mortality and HF exacerbations. One patient with LVEF below 25% died soon after LVSP due to ventricular fibrillation.

## Discussion

In this study, we found that LBBAP could significantly shorten the QRSd and improve the cardiac function in LBBB patients with LVEF > 35%. Compared with LVEF ≤ 35%, patients with LVEF > 35% showed lower risk of combined endpoint of death from any cause or hospitalizations for HF and better echocardiographic response to LBBAP.

Active measures have been taken on those patients with LVEF ≤ 35% and the mortality and hospitalization have been decreasing in recent years. However, patients with higher LVEF are not being treated positively and promptly at the same time. The data from the American Heart Association's Get With The Guidelines (GWTG)(22) which included 110,621 patients showed that preserved and borderline LVEF (>40%) accounted for about half of all HF hospitalizations and the number was on increase. And LVEF is recognized to be an independent predictor of mortality and morbility in HF patients (23). Patients with LVEF ranging from 36 to 45% still have higher risk of adverse outcomes. Further, Witt et al. (24) proved that in patients with LVEF between 35 and 50%, those with LBBB had poorer clinical outcomes than those without conduction disease in the long-term follow-up.

Recently, there have been some studies which aim to prove the effect of CRT in patients with LVEF > 35%. Fung et al. (25) and Foley et al. (26) both reported that CRT could improve cardiac function and reverse LV remodeling in small groups of HF patients with LVEF > 35%. And in PROSPECT trial (27), CRT improved the clinical composite score (CCS) and decreased left ventricular end-systolic volume (LVESV) similarly in patients with LVEF ≤ 35 and >35%. However, In REVERSE (28), the study discovered that in patients with LVEF > 30%, CCS was improving by CRT but of no significance. Besides, a statistically significant decrease of LV end diastolic volume index (LVEDVi) was only seen in patients with LVEF <30%. Reasons why REVERSE showed lower LV reverse remodeling than the other studies are unclear. Interestingly, a prospective, randomized, controlled, double-blinded study called MIRACLE EF study (29) which aimed to prove that CRT could achieve clinical benefit in patients combined with moderately reduced LVEF (36–50%) and LBBB with the minimum 24-month follow-up. However the study was stopped after 13 months due to poor recruitment and enrolling only 44 patients. Reasons are complicated but one reason may be in short of understanding the feasibility and necessity of preventive treatment in this population. Current studies show contradictory results and the sample size is too small to be convincing. Besides, there is a lack of non-CRT group comparison and multicenter, randomized study to reflect the clinical effect of CRT in HF patients with LVEF > 35%, especially in the presence of LBBB.

Since the population of patients with LBBB and LVEF from 36 to 50% has been on the increase and the prognosis of them is quite poor if any proper measure is taken, there exists the need to take effective interventions ahead of time. Except for CRT, LBBAP is another appropriate choice as a new strategy for physiological pacing to achieve electrical synchrony of LV with high success rate.

In recent years, there has been many articles to prove the safety and feasibility of LBBAP in LBBB patients. Zhang et al. (30) used to demonstrate that QRSd was significantly shortened with shorter interventricular mechanical delay by LBBAP. And in 2020, Guo et al. (12) made a comparison between LBBAP and biventricular pacing (BIV) and the study showed that LBBAP could restore electrical synchrony better and achieve greater improvement in echocardiographic and clinical outcomes. We can take LBBAP to be a feasible treatment as a rescue pacing method or as the primary pacing strategy for HF patients with CRT indications (13, 31).

Our research and previous studies have yielded similar results. Furthermore, in LVEF >35% group, the number of hyperresponders is more than that in LVEF ≤ 35% group. Meanwhile, in this group, more than half of the patients had LV restored [defined as return to NYHA I and LVEF > 50% (32)]. And LVEF > 35% group has higher R-wave amplitude, possibly because fewer people in this group have myocardial injury, fibrosis, or infarction, which contributes to better response to LBBAP. Besides, there may be a “sweet spot” (33) for LBBAP as well, just like CRT. If the ventricular function gets worse to a certain level, the myocardium is too “sick” to respond to any therapy. As a result of the decline in LVEF, adverse remodeling also progressed so that the cardiac function of patients is hard to return to normal.

In all the 36 patients recruited in our study, whether patients have LBBAP or LVSP, compared with LVEF ≤ 35% group, patients in LVEF > 35% group show significantly lower incidence of death from any cause or hospitalization for HF via LBBAP (*P* = 0.011). Besides, all-cause mortality is significantly lower in the LVEF > 35% group as well (*P* = 0.034). In our study, many of patients with primary endpoints had chronic kidney disease or persistent AF before procedure, both of which can accelerate the overall progression of HF independently. And in EAARN score (34), renal failure with GRF <60 mL/min/1.73 m^2^ was predictive of poor outcomes in patients treated with CRT. Besides, AF was associated with poorer survival in CRT patients despite the benefits of the therapy.

### Limitation

First, our study did not directly verify that LBBAP captured the cardiac conduction system by recording left bundle branch potential. Due to LBBB in most of patients, the potential could not be recorded in the conventional way; it could be achieved by double leads method, but this is not practical in regular clinical practice. In any event, our results of degree of narrowing QRSd and SPLVAT were comparable with other studies using direct left bundle branch potential recording (17–19). Thus the definition of LBBAP used in our study may include both left bundle branch pacing (LBBP) and LVSP. Nonetheless, LBBAP was supposed to have the same effect as LBBP. Second, the sample size was relatively small and follow-up was short-term. A large-scale randomized study with longer follow-up is necessary to clarify the role of LBBAP in these patients.

## Conclusion

LBBAP could significantly shorten QRS duration and improve cardiac function during medium-and-short term follow up in patients with LBBB and LVEF between 35 and 50%. The degree of echocardiographic and clinical improvement by LBBAP in these patients was better than those with LVEF ≤ 35%. Thus, LBBAP is a promising physiological ventricular pacing which could be an effective therapy for preventing the deterioration of cardiac function in early-stage HF patients.

## Data Availability Statement

The original contributions presented in the study are included in the article/Supplementary Material, further inquiries can be directed to the corresponding author/s.

## Ethics Statement

The studies involving human participants were reviewed and approved by the First Affiliated Hospital of Nanjing Medical University. The patients/participants provided their written informed consent to participate in this study.

## Author Contributions

All authors listed have made a substantial, direct, and intellectual contribution to the work and approved it for publication.

## Funding

This work was supported by the First Affiliated Hospital of Nanjing Medical University (JSPH-511C-2018-5).

## Conflict of Interest

The authors declare that the research was conducted in the absence of any commercial or financial relationships that could be construed as a potential conflict of interest.

## Publisher's Note

All claims expressed in this article are solely those of the authors and do not necessarily represent those of their affiliated organizations, or those of the publisher, the editors and the reviewers. Any product that may be evaluated in this article, or claim that may be made by its manufacturer, is not guaranteed or endorsed by the publisher.
